# A pharmacodynamic analysis of resistance trends in pathogens from patients with infection in intensive care units in the United States between 1993 and 2004

**DOI:** 10.1186/1476-0711-6-11

**Published:** 2007-10-01

**Authors:** Kathryn J Eagye, David P Nicolau, Shawn R Lockhart, John P Quinn, Gary V Doern, Gale Gallagher, Murray A Abramson

**Affiliations:** 1 Center for Anti-Infective Research and Development, Hartford Hospital, 80 Seymour Street, Hartford, CT 06102, USA; 2 Division of Infectious Diseases, Hartford Hospital, 80 Seymour Street, Hartford, CT 06102 USA; 3 Div. of Clinical Microbiology, University of Iowa Hospital and Clinics, 200 Hawkins Drive, Iowa City, IA 52242, USA; 4 John H. Stroger Hospital, 1900 West Polk Street, Chicago, IL 60612, USA; 5 Chicago Infectious Disease Research Institute, 1650 Harrison Street, Chicago, IL 60612, USA; 6 Rush University Medical Center, 1650 Harrison Street, Chicago, IL 60612, USA; 7 Merck Research Laboratories, Merck & Co., 126 E Lincoln Ave., Rahway, NJ 07065, USA

## Abstract

**Background:**

Increasing nosocomial pathogen resistance to available antimicrobial agents is of growing concern. While higher MICs can diminish antimicrobial effectiveness, dose adjustments often mitigate this effect. This study's objective was to ascertain whether MICs among major pathogens in the ICU to several commonly used agents have increased enough to significantly impact their ability to achieve bactericidal effect.

**Methods:**

Cefepime, ceftriaxone, imipenem and piperacillin-tazobactam MICs were determined with 74,394 Gram-negative bacilli obtained from ICU patients with various infections in the US between 1993 and 2004. Results were grouped into four 3-year periods. The predicted cumulative fraction of response (CFR) was estimated based on patient-derived pharmacokinetic values and Monte Carlo simulation. Trends in CFR over the four study periods were assessed using the Cochran-Armitage test. The primary analysis included all organisms combined; *Pseudomonas aeruginosa *and *Acinetobacter *species were also evaluated individually.

**Results:**

In the primary analysis, imipenem 500 mg q6h showed CFRs from 87% to 90% across all four study periods, with a trend toward slightly improved bactericidal target attainment (p < 0.01). CFRs for cefepime 2 g q12h and piperacillin-tazobactam 4.5 g q6h both declined by 2% (p < 0.01 and p < 0.05, respectively), reflecting upward shifts in the underlying MIC distributions. Ceftriaxone had <52% CFR for all regimens in all periods, with no significant trend. Against *P. aeruginosa*, significant declines in CFR were seen for (range, p-value): imipenem 1 g q8h (82%–79%, p < 0.01), cefepime 1 g q12h (70%–67%, p < 0.01), cefepime 2 g q12h (84%–82%, p < 0.05), piperacillin-tazobactam 3.375 g q6h (76%–73%, p < 0.01), piperacillin-tazobactam 4.5 g q8h (71%–68%, p < 0.01), and piperacillin-tazobactam 4.5 g q6h (80%–77%, p < .01). Against *Acinetobacter *spp., all regimens of imipenem, cefepime and piperacillin-tazobactam showed significant declines in CFR over time (p < 0.01).

**Conclusion:**

Our observations suggest that as a result of increasing antimicrobial resistance among ICU pathogens in the US, drug effectiveness, assessed as a function of individual agents' ability to attain pharmacodynamic targets, has declined, especially with *P. aeruginosa *and *Acinetobacter *spp. Cefepime 2 g q8h and imipenem were the most potent agents against these species, respectively. More aggressive dosing of all of the agents characterized could preserve their clinical utility, but this must be balanced with safety and tolerability issues by the physician.

## Introduction

Surveillance studies have revealed increasing rates of resistance among bacteria commonly implicated in serious hospital infections; resistant pathogens are associated with higher mortality rates than are susceptible organisms[1,2]. As the pipeline of new antimicrobial agents for Gram-negative pathogens shrinks, the longevity of existing compounds becomes a matter of primary concern[3].

Our earlier work has shown that pharmacokinetic/pharmacodynamic (PK/PD) modeling based on Monte Carlo simulations can be used reliably to predict the ability of antimicrobial regimens to achieve maximum bactericidal effect against organisms implicated in nosocomial infections[4]. Further, we have demonstrated that alterations in dose can extend the coverage of many current compounds[5,6].

Using Monte Carlo analyses, the objective of the current study was to use PK/PD modeling to assess the profile of activity of four antimicrobial agents commonly used to treat serious infections – imipenem, ceftriaxone, cefepime and piperacillin-tazobactam – versus a large collection of bacteria recovered from patients in the intensive care unit (ICU) setting in the United States between 1993 and 2004. These organisms had been characterized as part of the Merck Intensive Care Unit Surveillance Survey (ISS) Program. Secondly, we attempted to determine if the activity profile of any of these antimicrobial agents had diminished over time. A third objective was to determine the effect of dose selection on the activity profile of the agents.

## Methods

This investigation employed Monte Carlo simulation techniques to estimate the relative probability that various antimicrobial agents would achieve maximally effective (i.e. bactericidal) exposures against isolates of Gram-negative bacilli recovered from patients with infection in the ICU. A PK model was developed for each compound and then used in a simulation to incorporate patient variability. The model, the input parameters, and the simulation technique are described below. The following antibiotic regimens were examined (administered as 30-minute intravenous infusions): cefepime 1 gram (1 g) every twelve hours (q12h), 2 g q12h and 2 g q8h; ceftriaxone 1 g q24h and 2 g q24h; imipenem 500 mg q6h and 1 g q8h; and piperacillin/tazobactam 3.375 g q6h, 4.5 g q8h and 4.5 g q6h.

### Microbiology

MICs for bacterial isolates used in the analysis were obtained from the ISS Program from 1993 to 2004. A total of 44 different species were characterized. The 11 most commonly recovered species (which account for 94% of the tested population) are displayed in Table 1. For each compound, MIC results from all organisms tested were grouped to form one MIC frequency distribution for four intervals of three years each: 1993–1995, 1996–1998, 1999–2001, and 2002–2004. The MIC distributions were incorporated into the analysis as described in the following section, "Cumulative Fraction of Response." This scenario (all isolates included) was modeled in the primary analysis. Additionally, MIC distributions for two individual species, *P. aeruginosa *and *Acinetobacter *spp, were similarly divided into four intervals of the same 3-year periods. These groupings were used for sub-analyses of those species.

**Table 1:** Eleven (11) most commonly recovered species (constituting 94% of the tested population) by frequency extracted from the Merck ICU Surveillance Program from 1993 to 2004 (Total of all isolates: 74,394).

Organism	Number (%)	Cumulative frequency
*Pseudomonas aeruginosa*	16,482 (22)	22.2%
*Escherichia coli*	13,961 (19)	40.9%
*Klebsiella pneumoniae*	10,571 (14)	55.1%
*Enterobacter cloacae*	6,779 (9)	64.2%
*Acinetobacter *spp.	4,642 (6)	70.5%
*Serratia marcescens*	4,112 (6)	76.0%
*Enterobacter aerogenes*	3,307 (4)	80.5%
*Stenotrophomonas maltophilia*	3217 (4)	84.8%
*Proteus mirabilis*	3,011 (4)	88.8%
*Klebsiella oxytoca*	2,018 (3)	91.5%
*Citrobacter freundii*	1,483 (2)	93.5%

### Pharmacokinetic model

A two-compartment multiple dose model was developed to determine the 24-hour concentration-time profile at steady-state for each drug regimen. PK parameters were used as input variables, and included total body clearance (*CL*_*T*_), volume of the central compartment (V_*c*_), and the microtransfer rate constants between the central and peripheral compartments (*k*_12 _and *k*_21_). Estimates of unbound fraction for each drug (*f*_*u*_) were also used as inputs.

PK parameters for each compound under study were obtained from previously published population PK studies in critically ill patients, except for *f*_*u*_, which was derived from the package insert for each drug; the mean and standard deviation for each of these parameters have been previously published[5,7]. Covariance matrices were either reported in or were calculated from these studies and were applied to the input pharmacokinetic variables used in the simulations.

### Monte Carlo simulation

A 5,000 trial Monte Carlo simulation (Crystal Ball 2000, Decisioneering, Denver, CO, USA) was conducted for each antimicrobial regimen using the pharmacokinetic model in order to determine the regimen's probability of target attainment (PTA) profile. PTA is the probability that the regimen will meet or exceed a pre-defined pharmacodynamic target at a given MIC dilution[8]. Here, PTA is the proportion of the 5,000 trials in each simulation that achieved the target level of a PD index at each MIC in doubling dilutions from 0.008 to 128 μg/ml.

The target indices were selected based on the PD properties (i.e., time-dependent or concentration-dependent killing) of the compound. As the four compounds modeled here exhibit time-dependent killing, the proportion of the dosing interval during which the concentration of free (unbound) drug remains above the MIC is the appropriate PD index[9]. For the carbapenems (including imipenem), 40% *f*T>MIC was considered bactericidal; 50% *f*T>MIC was considered bactericidal for piperacillin-tazobactam and for the cephalosporins[9,10]. The PTA profile was then determined for each regimen.

### Cumulative Fraction of Response

CFR is the probability that the regimen will attain its PD index against the specific population of organisms characterized in the ISS Program. Each regimen's simulation-derived PTA is multiplied by the percentage of isolates found at each MIC dilution; the sum of these products is the CFR[8]. For each scenario (all isolates in the primary analysis or an individual species in the sub-analyses), this process was applied to four MIC distributions, one from each three-year interval, to obtain the CFR for each regimen during each interval.

A CFR of 90% was considered to be the threshold for achieving reliable empiric therapy[11]. Confidence intervals were calculated around each CFR result to provide a measure of statistical significance; however, it is important to consider these results in light of clinical significance as well. The CFR for each interval can be compared to illustrate trends in the ability of each regimen to achieve its best killing effect. A p-value ≤ 0.05 indicates a statistically significant trend across the four periods, where lower CFRs in later periods reflect a decline in activity (higher CFRs would indicate an improvement). Confidence intervals around each CFR probability were calculated at α = 0.05 using the Newcombe-Wilson method without correction for continuity[12]. Trends in cumulative fraction of response for each regimen were assessed using the Cochran-Armitage test for trend (SAS version 9.0, Cary, NC, USA).

## Results

Results of the primary analysis are presented in Table 2. Against all species aggregated, imipenem demonstrated a trend toward slightly improving activity over the four periods, and had predicted responses near 90%. Cefepime displayed a decreasing activity profile over time, though still showing generally high predicted responses (80%–90%, depending on dosing regimen). Ceftriaxone had relatively low predicted response rates (< 52%) across all periods, and no significant trend was detected. Piperacillin-tazobactam 4.5 g q6h displayed the highest predicted response among piperacillin-tazobactam regimens (80–78%), with a trend toward decreasing activity. The less aggressive piperacillin-tazobactam regimens (4.5 g q8h and 3.375 g q6h) displayed lower CFRs and were stable to trend.

**Table 2:** Cumulative fraction of response for various drug regimens against MICs collected from the Merck ICU Surveillance Study (ISS) Program, in four time intervals.

Antimicrobial agent:	CFR^a ^(%)				
	
Time interval:	1993–1995^b^	1996–1998	1999–2001	2002–2004	p-value
Imipenem 500 mg q6h	87.0	87.7	88.2	90.6	<.0001
Imipenem 1 g q8h	86.8	87.3	87.9	90.2	<.0001
Ceftriaxone 1 g q24h	32.5	30.9	32.3	31.5	.6128
Ceftriaxone 2 g q24h	51.6	49.2	51.2	50.0	.3726
Cefepime 1 g q12h	-	80.6	80.4	78.6	.0118
Cefepime 2 g q12h	-	86.8	87.5	84.8	.0027
Cefepime 2 g q8h	-	91.8	93.5	89.9	.0006
Pip-Tazo 3.375 g q6h	-	76.6	77.3	75.1	.0667
Pip-Tazo 4.5 g q8h	-	72.1	72.9	70.9	.1569
Pip-Tazo 4.5 g q6h	-	79.4	79.9	77.5	.0175

^a^CFRs were calculated at 40% *f*T>MIC for imipenem and 50% *f*T>MIC for the other compounds.

^b^No data for cefepime or piperacillin-tazobactam (pip-tazo) was provided for 1993–1995.

Sub-analyses were conducted on two species which have been shown in the literature to be highly pathogenic and whose resistance is of current concern: the results for *P. aeruginosa *and *Acinetobacter *spp. are displayed in Tables 3 and 4, respectively. Against both species, all active agents showed significant declines in predicted CFR over time. For *P. aeruginosa*, the greatest activity was observed with higher doses of cefepime (particularly 2 g q8h). Against *Acinetobacter *spp., imipenem displayed the most favorable activity profile, with predicted CFRs >20% higher than cefepime and piperacillin-tazobactam.

**Table 3:** Cumulative fraction of response for various drug regimens against *P. aeruginosa *MICs collected from the Merck ICU Surveillance Study (ISS) Program, in four time intervals.

Antimicrobial agent:	CFR^a ^(%)				
	
Time interval:	1993–1995^b^	1996–1998	1999–2001	2002–2004	p-value
Imipenem 500 mg q6h	81.6	81.9	79.5	78.5	<.0001
Imipenem 1 g q8h	81.9	82.0	79.8	78.7	<.0001
Ceftriaxone 1 g q24h	1.4	1.4	1.4	1.3	.6217
Ceftriaxone 2 g q24h	3.2	3.2	3.1	2.8	.2903
Cefepime 1 g q12h	-	69.9	68.3	67.2	.0035
Cefepime 2 g q12h	-	83.5	82.9	81.8	.0240
Cefepime 2 g q8h	-	92.2	92.7	91.2	.0842
Pip-Tazo 3.375 g q6h	-	75.9	74.0	73.3	.0024
Pip-Tazo 4.5 g q8h	-	70.8	69.0	68.4	.0095
Pip-Tazo 4.5 g q6h	-	79.6	77.5	76.7	.0004

^a^CFRs were calculated at 40% *f*T>MIC for imipenem and 50% *f*T>MIC for the other compounds.

^b^No data for cefepime or pip-tazo was provided for 1993–1995.

**Table 4:** Cumulative fraction of response for various drug regimens against *Acinetobacter *spp. MICs collected from the Merck ICU Surveillance Study Program (ISS), in four time intervals.

Antimicrobial agent:	CFR^a ^(%)				
	
Time interval:	1993–1995^b^	1996–1998	1999–2001	2002–2004	p-value
Imipenem 500 mg q6h	94.5	91.5	84.8	84.6	<.0001
Imipenem 1 g q8h	94.2	92.0	86.2	86.1	<.0001
Ceftriaxone 1 g q24h	2.4	1.8	1.9	1.9	.1651
Ceftriaxone 2 g q24h	6.7	5.1	5.2	4.9	.0005
Cefepime 1 g q12h	-	50.8	45.2	35.4	<.0001
Cefepime 2 g q12h	-	65.8	63.1	49.7	<.0001
Cefepime 2 g q8h	-	78.5	80.3	64.4	<.0001
Pip-Tazo 3.375 g q6h	-	56.4	51.3	43.6	<.0001
Pip-Tazo 4.5 g q8h	-	51.9	47.4	40.5	<.0001
Pip-Tazo 4.5 g q6h	-	61.0	55.3	46.6	<.0001

^a^CFRs were calculated at 40% *f*T>MIC for imipenem and 50% *f*T>MIC for the other compounds.

^b^No data for cefepime or pip-tazo was provided for 1993–1995.

## Discussion

We examined nosocomial isolates collected from ICUs in the United States during a multi-year surveillance study in order to assess the performance of antibiotics commonly used to treat infections in this setting, and to identify any trends in antimicrobial performance over time. Four broad-spectrum agents were studied: cefepime, ceftriaxone, imipenem and piperacillin-tazobactam. We used PK/PD principles to predict the likelihood that each compound and dose would achieve its maximum bactericidal effect against the tested population of organisms, as measured by cumulative fraction of response. The Monte Carlo simulation technique enables us to predict the microbiological performance of different regimens while accounting for the variance in PK characteristics that such regimens are likely to encounter in critically ill patients. It should be noted that microbiological success is but one part of clinical success in treating infection, as many factors – co-morbidities, immunocompetence, etc. – contribute to the ultimate recovery of a patient. However, we have found that simulation-predicted microbiological response does indeed correlate with clinical response[13]. As such, identifying trends in probable microbiological success can provide useful insight into the clinical implications of changing resistance. We found moderate to good predicted responses (80% CFR or better) to all species aggregated as a group for three of the four compounds examined, with low response rates predicted for ceftriaxone. Response rates were dose-dependent, with more aggressive doses yielding greater predicted response. As to trend, imipenem's ability to achieve bactericidal effect showed a slight statistical improvement over time, whereas the effect of shifting MIC distributions on the other compounds was reflected in stable or worsening activity.

The examination of antibiotic performance against the entire cohort of isolates in the ISS Program is useful as a broad evaluation of continued efficacy to pathogens encountered in the ICU; however, clinically significant trends in any one species may be masked by the trend (or lack thereof) in the overall group. For this reason, the study also examined two species individually: *P. aeruginosa *and *Acinetobacter *species. Both species are implicated in a variety of nosocomial infections (including pneumonia, bacteremia, skin infections and others), and reports of multi-drug resistant strains have been increasing[14]. *P. aeruginosa *was the most prevalent species in the ISS Program dataset, at 22% of tested isolates. This species is an important nosocomial pathogen not only because of its frequency, but also because it possesses intrinsic resistance to many antimicrobial agents, and has the ability to acquire both plasmid-mediated and chromosomal resistance genes[15]. *Acinetobacter *spp. was the fifth most prevalent pathogen in the dataset (at 6%), but was modeled individually because high levels of resistance have been reported in Latin America and in some regions of the United States[1,16]. Multi-drug resistance in these two species is associated with increased mortality, and has become sufficiently problematic in some locations that alternative therapy using older and more toxic agents such as polymyxins is sometimes considered the best option [17-19].

Against *P. aeruginosa*, cefepime, imipenem and piperacillin-tazobactam showed statistically significant declines in CFR over time for all regimens except cefepime 2 g q8h. This regimen also was clearly the most potent, with predicted CFRs >90%, where every other regimen had predicted CFRs <80% by the last time period. This suggests that, while more aggressive dosing may enable a compound to kill more reliably (i.e., with a greater probability of success), this effect no longer holds once the potency of the agent has sufficiently eroded. Overall, the magnitude of the CFR declines for all the regimens over the 12-year period of the study remains small, reflecting a steady, although moderate, upward shift in the distribution of MICs.

Against *Acinetobacter *spp., statistically significant declines in CFR were observed for all regimens of cefepime, imipenem and piperacillin-tazobactam. Further, the magnitude of the decline across periods is much greater than that observed against *P. aeruginosa *– on the order of 10%–15% decline in CFR against the former species versus 2–3% versus the latter. While both declines show statistically significant trends, whether or not the low single digit declines against *P. aeruginosa *are clinically significant is debatable – that is, these declines in effectiveness are probably noticed in some institutions more than others, to the extent that the higher MICs observed here are not evenly distributed among hospitals. However, the larger declines in predicted efficacy against *Acinetobacter *are of great enough magnitude that a clinical effect may commonly be seen. Indeed, cefepime and piperacillin-tazobactam were predicted to have such low CFRs (<50% for all regimens except cefepime 2 g q8h, with a predicted CFR of 65%) as to be of little use clinically. Imipenem, while showing declines in CFR over the four time periods, is the only modeled agent with a high enough probability of achieving its best bactericidal effect to be relied upon clinically. These results reflect a species with rapidly increasing MICs to the modeled compounds.

## Conclusion

The MIC distributions of ICU pathogens to these commonly prescribed compounds have increased significantly over time, and their effectiveness as measured by ability to attain PD targets has declined. Statistically significant declines in effectiveness were noted against *P. aeruginosa *and *Acinetobacter *spp. for all regimens analyzed except for ceftriaxone, which has no activity to these organisms. Against *P. aeruginosa*, cefepime 2 g q8h remains a potent choice for therapy; the therapeutic utility of imipenem and piperacillin-tazobactam can be maximized by aggressive dosing. Against *Acinetobacter *spp., declines in effectiveness are statistically (and likely clinically) significant. Among the modeled agents, only imipenem remains a viable therapeutic option against this organism; more aggressive dosing of the other compounds does not bring current effectiveness within range of earlier clinical reliability, suggesting that an impact on outcomes may be observed.

## Competing interests

KJE and SL have no competing interests to declare. DPN, JPQ and GVD have received research grants from and have acted as consultants to the study sponsor. GG and MAA are employees of the study sponsor.

## Authors' contributions

KJE performed the statistical analyses and drafted the manuscript. DPN conceived of the study, participated in its design, and helped to draft the manuscript. SL, JPQ and GVD contributed to the design of the study and the interpretation and analysis of data, and GVD and JPQ served as study directors. GG and MAA coordinated and executed the Intensive Care Unit Surveillance Study Program, and managed the data. All authors read and approved the final manuscript.
